# CaCO_3_ loaded lipid microspheres prepared by the solid-in-oil-in-water emulsions technique with propylene glycol alginate and xanthan gum

**DOI:** 10.3389/fnut.2022.961326

**Published:** 2022-08-22

**Authors:** Gongwei Li, Yicong Zhao, Jie Zhang, Jia Hao, Duoxia Xu, Yanping Cao

**Affiliations:** Beijing Advanced Innovation Center for Food Nutrition and Human Health (BTBU), School of Food and Health, Beijing Higher Institution Engineering Research Center of Food Additives and Ingredients, Beijing Technology and Business University, Beijing, China

**Keywords:** CaCO_3_, S/O/W calcium-lipid emulsions, propylene glycol alginate (PGA), xanthan gum (XG), physical stability, structural characteristic, interaction

## Abstract

Calcium carbonate (CaCO_3_) is difficult to deliver in food matrices due to its poor solubility. In this work, CaCO_3_ powders were encapsulated into Solid-in-Oil-in-Water (S/O/W) emulsions to fabricate delivery systems. The impact of the concentrations of propylene glycol alginate and Xanthan gum (PGA-XG) complexes on the physical stability and structural characteristics of S/O/W calcium-lipid emulsions microspheres were studied. The S/O/W calcium-lipid emulsions were characterized by the particle size, zeta potential, physical stability, and apparent viscosity. The S/O/W calcium-lipid emulsion has higher physical stability (including 6-week storage at 4°C), smaller mean particle size (7.60 ± 1.10 μm), and higher negative zeta-potential (45.91 ± 0.97 mV) when the concentration of PGA-XG complexes was 0.8 wt%. Moreover, Confocal laser scanning microscopy (CLSM) images confirmed that the CaCO_3_ powders were encapsulated in the O phase. Transmission electron microscopy (TEM) showed that S/O/W calcium-lipid emulsion was spherical. The X-ray diffraction (XRD) analysis further confirmed that CaCO_3_ was loaded in the S/O/W calcium-lipid emulsion as an amorphous state. The formation mechanism of S/O/W calcium-lipid microspheres was studied by Fourier transform infrared spectroscopy (FTIR) and Raman spectrum analysis. This study provided new ideas that accelerate the creation of a novel type of calcium preparation with higher quality utilization.

## Graphical Abstract

Schematic diagram of the construction and characterization of S/O/W calcium-lipid microspheres. 
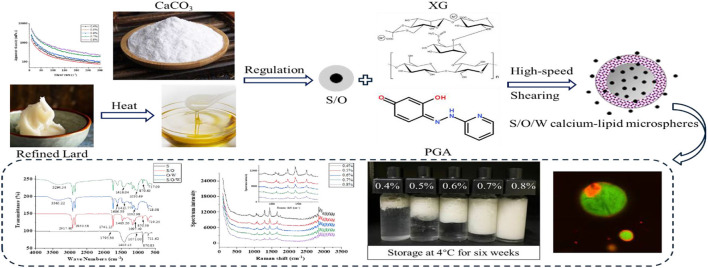

## Introduction

The human body is composed of more than 80 elements, of which calcium is the most abundant metal element that participates almost in various physiological activities of the human body (1). Calcium balance is a significant condition for maintaining the normal operation of the human body. Calcium deficiency seriously affects health. In daily life, infantile convulsions, hand-foot convulsions, maternal cramps, and osteoporosis in the elderly are all caused by calcium deficiency. The global calcium map reveals that many countries have low average calcium intake (2). Therefore, many people pay more and more attention to calcium supplementation in daily life, which also makes calcium to become a research focus. Some investigators have done numerous studies on materials that can promote bone regeneration. Elsheikh et al. (3) converted calcium sulfate into calcium carbonate (CaCO_3_), then converted CaCO_3_ into carbonate apatite in different interconnected pore volumes. It was found that interconnected pores of carbonate apatite promote rapid bone regeneration. Meng et al. (4) reported that β-tricalcium phosphate/calcium phosphate cement and chitosan microsphere/calcium phosphate cement could promote proliferation and differentiation of osteoblasts. Chen et al. (5) used CaCO_3_ to prepare multistage-structured microspheres that are pH responsive to treat osteomyelitis.

Calcium-strengthening sources are mainly divided into soluble calcium and insoluble calcium. Due to the strong interaction between soluble calcium and proteins, protein bridging flocculation is caused, resulting in the thermal instability of food systems, such as dairy products. Insoluble calcium does not cause thermodynamic instability of protein, but its suspension stability is poor and easy to precipitate. As reported, CaCO_3_, calcium citrate, calcium fumarate etc., have no significant difference in absorption *in vivo* (6). The CaCO_3_ has high calcium content (40 wt%) and cheaper price. Therefore, the usually utilized strengthening calcium is mainly CaCO_3_ (7). In the field of food, CaCO_3_ is mainly added in milk powder, biscuit, candy etc. In addition, commercially available calcium preparations are mostly CaCO_3_, such as caltrate. However, most of the calcium preparations sold in the market are tablets, which are not conducive to the consumption of infants, the elderly, and persons with chewing difficulties. At the same time, there are still problems, such as poor absorption and easy constipation. Therefore, the design of new delivery systems for food nutrients to improve the solubility and bioaccessibility is the aim of developing the healthy food.

Microemulsions, liposomes, nanoemulsions, emulsions, microgels, filled microgels, colloidosomes, multiple emulsions, microclusters, biopolymer nanoparticles, etc., are commonly utilized in the food delivery system (8–11), which may be utilized to boost the digestibility, absorptivity, and stability of the available lipophilic and hydrophilic nutrients (12). There are little literature about the carrier of solid nutrients e.g., mineral elements. To achieve the three-phase homogenization of solid (S), oil (O), and water (W) in the food production process, it is essential to seek the novel carrier of the transfer system.

In the process of dairy production, insoluble calcium salt is usually added directly, in which the micronized calcium powder particles disperse freely. As reported, this dispersion system is thermodynamically unstable, and the gravity and dispersal ability affect the distribution of calcium salt particles (13). According to Stokes law, the sedimentation rate of particles is related to the density of particles and the viscosity of the medium. In addition, particles with different granularity in the dispersion system have irregular Brownian motion, and the disorderly collision of molecular thermal motion causes particle agglomeration. The smaller the particle size, the larger the surface area, and the higher the surface energy, the easier the particles are to agglomerate. Therefore, the critical operation in liquid food is to solve the poor solubility of calcium salt.

Microsphere carrier with specific structure was constructed based on Solid-in-Oil-in-Water (S/O/W) techniques, which can embed probiotics and enzyme preparations, realize the controlled release of embedded solid components, and improve the physicochemical stability and bioaccessibility (14–17). This technique has the advantages of concise steps and lower production costs to deliver solid nutrients. In recent years, proteins and polysaccharides as natural emulsifiers have attracted wide attention from scientists and food manufacturers. Xanthan gum (XG) is an anionic extracellular polysaccharide engendered by Xanthomonas *campestris* through aerobic fermentation of sugar (18, 19). XG, as a hydrophilic polymer, was easy to form hydrogen bonds with water. However, XG was difficult to combine with oil for the reason that it does not have significant hydrophobic bond (20). Propylene glycol alginate (PGA) is a linear polysaccharide with high molecular weight (21). Due to the hydrophobic property of the propylene glycol group, the PGA molecule has interfacial activity, which can be foaming and emulsifying (22, 23). Therefore, the PGA-XG complexes can change the surface activity, thereby improving the emulsion stability.

Herein, we focused on the formation of S/O/W calcium-lipid microspheres emulsions using PGA and XG polysaccharides as natural emulsifiers. In this study, we systematically investigated the particle size, instability index, zeta potential, and the apparent viscosity of the S/O/W calcium-lipid microspheres prepared by high-speed shear. In addition, Confocal laser scanning microscopy (CLSM), Transmission electron microscopy (TEM), Fourier transform infrared spectrometer (FTIR), laser Raman spectrometer, and X-ray diffractometer (XRD) were used to examine the microstructure, the interaction, and structural characteristic of S, O, and W phases in S/O/W calcium-lipid microspheres. This provides a theoretical and technical basis for the development of a new mineral transfer system. This work might hence offer fresh ways which speed the product of a novel type of calcium preparation with higher quality utilization.

## Materials and methods

### Materials

Light CaCO_3_was acquired from Shuang Teng Industrial Co., Ltd. (Zhengzhou, China). The oil phase used for the formulation of the S/O/W emulsion was refined lard, which was bought from Xincheng Jinluo Meat Products Group Co., Ltd. (Linyi, China). XG (CAS#11138-66-2, purity > 99.5%) was procured from Beijing Biotopped Science and Technology Co., Ltd. (Beijing, China). PGA (CAS#9005-37-2, purity > 98%) was obtained from Yuanye Bio-Technology Co., Ltd. (Shanghai, China). Nile Red was provided by Sigma-Aldrich Co. (St. Louis, MO, USA). Rhodamine B (RB) was purchased from Shanghai Aladdin Biochemical Technology Co., Ltd. (Shanghai, China). Ultrapure water was used to prepare all solutions. It was prepared in a Milli-Q water purification system.

### Preparation of S/O/W calcium-lipid microspheres

Different concentrations of XG solutions (0.4, 0.5, 0.6, 0.7, and 0.8 wt%) and PGA solutions (0.4, 0.5, 0.6, 0.7, and 0.8 wt%) were prepared separately by dissolving XG and PGA powder in phosphate buffer (1.0 mM, pH 6.0) by stirring for 2 h at 50°C followed by 24 h incubation at 4°C until it was fully dissolved. The preparation of W phase was mixed with equal concentrations of XG solutions and PGA solutions at the ratio of 6:4.

The preparation of the S/O/W calcium-lipid emulsions was performed similar to our former study (24) with modifications of the S/O phase preparation conditions. The refined lard was heated at 50°C water bath until fully melted to obtain the O phase. The S/O phase was prepared by the addition of 10% of the S phase (CaCO_3_) to 100% of the O phase. The S/O phase was pre-dispersed using a mixer (ULTRA TURRAX T25 digital, IKA, Staufen, Germany) for 60 s at the speed of 9,500 rpm followed by 1 h magnetic stirring at 30°C to ensure uniform distribution of the S phase in the O phase. The mixture of 5% of S/O phase added to 95% of W phase was subjected to high-speed shearing at 15,000 rpm for 5 min to acquire S/O/W calcium-lipid emulsions.

### Particle size and zeta potential measurements

The dimension of S/O/W calcium-lipid emulsions was measured using an SDC-Microtrac S3500 (Microtrac, Montgomery Ville, USA) laser diffraction equipment. The sample was transported to the measuring unit with water (refractive index 1.33) as a medium, and the sample was subjected to wet measurement (24, 25). Each sample was repeatedly measured thrice. Data acquisition and analysis were carried out using Microtrac's FLEX11 software.

The zeta potential of S/O/W calcium-lipid emulsions was studied by Zetasizer Nano-ZS90 (Malvern Instruments, Worcestershire, UK). To avert the effect of multiple scattering, we utilized a 1.0 mM buffer solution with the same pH as the samples to dilute S/O/W calcium-lipid emulsions to about 0.0125 % of the oil content before measurements. According to the theory of Smoluchowski, the data of the samples were equilibrated at 25°C for about 120 s and were calculated over 11 continuous readings. The refractive indices of the continuous phase and dispersed phase were set as 1.33 and 1.45, respectively.

### Physical stability measurements

The stability of fresh S/O/W calcium-lipid emulsions was determined by LUMiSizer (LUM GmbH, Berlin, Germany), which can accelerate the change of sample instability. The measurement parameters were as follows: injection volume, 0.4 mL; wavelength of the detection light, 865 nm; temperature, 25 °C; profile lines, 255; rotational speed, 4,000 rpm; light factor, 1.00; time interval, 60 s; total duration, 4 h 15 min. The instability index from the meniscus to the bottom of the emulsion was calculated.

Meanwhile, the 20 ml fresh S/O/W calcium-lipid emulsions formed by different concentrations of PGA-XG complexes were stored at 4°C and were monitored using visual observation for 6 weeks.

### Apparent viscosity measurements

The apparent viscosity of S/O/W calcium-lipid emulsions as prepared above were measured by continuous shear test using HAAKE rheometer (Mars IQ Air, Karlsruhe, Germany). Using the CC25 DIN rotor, the shear rate ramps were conducted from 1 to 200 s^−1^ at 25°C.

### Confocal laser scanning microscopy

Confocal laser scanning microscopy (FV3000, Olympus, Japan) was used to observe the microstructure of S/O/W calcium-lipid emulsions. Before emulsification, the O phase (Refined lard) was dyed by Nile Red, while the S phase (CaCO_3_) was stained with RB (26). Fluorescence from RB and Nile Red was excited at 543 nm and 488 nm, respectively. S/O/W calcium-lipid emulsions ought to be diluted 10-fold before using a 10 × eyepiece under 60 × objective lens (oil immersion) observation. The resolution of the CLSM images of 1024 × 1024 pixels were read and analyzed by the software of the instrument (FV10-ASW 4.1 Viewer, Olympus, Tokyo, Japan).

### Transmission electron microscopy

Transmission electron microscopy (JEM 1200EX, JEOL, Japan) was used to observe the morphological characteristics of S/O/W calcium-lipid emulsions prepared by 0.8 wt% concentration of PGA-XG complexes at an operating voltage of 100 kV. Samples ought to be diluted 10-fold before using phosphotungstic acid negative staining.

### X-ray diffraction

The crystalline structure of CaCO_3_ (S) and the lyophilized XG and PGA mixed solution (W), the lyophilized O/W emulsions without CaCO_3_, and the lyophilized S/O/W calcium-lipid emulsions were measured using an XRD (D8 Advance, Bruker, Germany). The scanning speed used was 2°/min in the range of 5°-50° diffraction angle (2θ). The acceleration voltage was 40 kV and the tube current was 40 mA.

### Fourier transform infrared spectroscopy

Fourier transform infrared spectra of the S phase, S/O phase, O/W phase, and S/O/W phase were measured by using an FTIR spectrometer (Nicolet iS10, Thermo Nicolet, USA). Freeze-dried samples were mixed with 100 mg of KBr powder, which were ground to a fine powder and pressed to a pellet. The spectra were obtained after 64 scans at the wavenumbers ranging from 4,000 to 400 cm^−1^ with a 4 cm^−1^ resolution.

### Raman analysis

The Raman spectra of CaCO_3−_loaded S/O/W calcium-lipid emulsions and four components (i.e., S phase, O phase, S/O phase, and W phase) of the emulsion were detected by a Raman microspectroscopy system (inVia-Qontor, Renishaw, UK). Each spectrum of the sample was collected at the laser excitation wavelength of 785 nm, laser power of 100 mW, 3 scans, and exposure time of 10 s. The Raman spectra of each sample were analyzed in the regions of 100–3,300 cm^−1^ and performed in duplicate.

### Statistical analysis

All tests were carried out at least in triplicate and the results were expressed as means ± standard deviations of the measurements. The Origin 8.5 software and SPSS 17.0 statistical analysis system (SPSS Inc., Chicago, IL) were used to draw the plots and to analyze the data, respectively. Statistical analysis was performed on the obtained results using one-way analysis of variance (ANOVA) analysis and Duncan multiple range test. The significance level (P) was set at 0.05.

## Results and discussion

### Particle size and size distribution of S/O/W calcium-lipid emulsions

**Figure 1:**
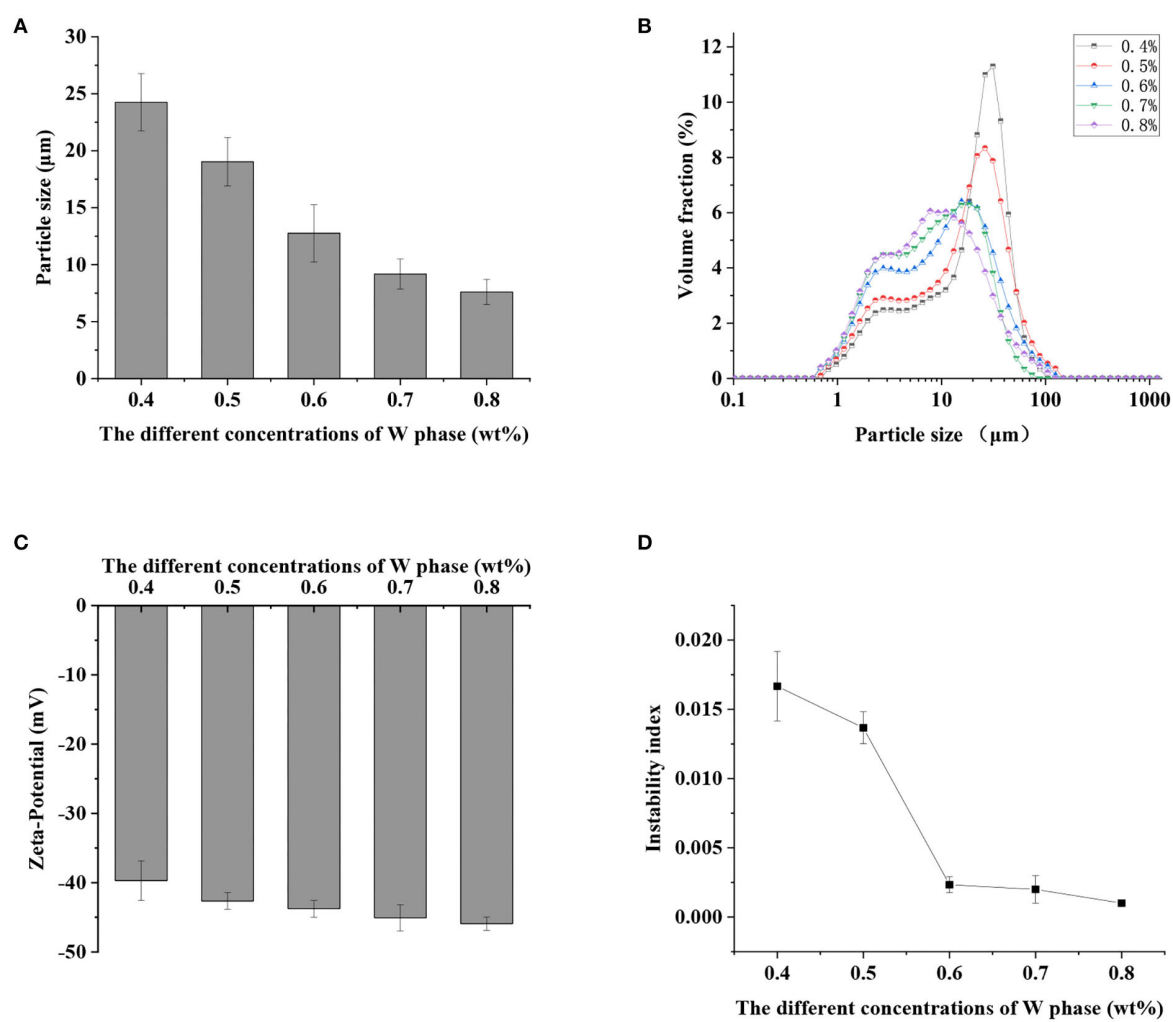
Effect of the different concentrations of propylene glycol alginate and Xanthan gum (PGA-XG) complexes (0.4, 0.5, 0.6, 0.7, and 0.8 wt%) on the particle size **(A)**, particle size distribution **(B)**, zeta potential **(C)**, and instability index **(D)** of S/O/W calcium-lipid emulsions.

The average particle size and particle size distribution of S/O/W calcium-lipid emulsions could be used to characterize and monitor the stability of the emulsion system. The average particle size and droplet size distribution of S/O/W calcium-lipid emulsions formed by different concentrations of PGA-XG complexes are shown in Figures 1A,B, respectively. The complexes of XG solutions and PGA solutions in the ratio of 6:4 formed an interface layer on the droplet surface, which increased the spatial repulsion between adjacent droplets and reduced van der Waals force between droplets (27). The instability of S/O/W calcium-lipid emulsions was prevented. It could be seen from Figure 1A that the average particle size decreased gradually with the increase in PGA-XG complexes concentration ranging from 0.4 to 0.8 wt%. The minimum average particle size of the S/O/W calcium-lipid microspheres was 7.60 ± 1.10 μm. Meanwhile, it could be observed from Figure 1B that the droplet size distribution moves to the left and becomes narrower gradually with the increase in PGA-XG complexes concentration (0.4–0.8 wt%). The reason might be that with the increase in the concentration of PGA-XG complexes, the emulsification ability became stronger, the number of S/O/W calcium-lipid emulsion particles increased, and the single-particle size decreased.

### Zeta potential of S/O/W calcium-lipid emulsions

According to Derjaguin-Landau-Verwey-Overbeek (DLVO) theory, the stability of the colloidal particle suspension depends on the equilibrium between the electrostatic interaction and the van der Waals interaction between particles (28). Zeta potential was utilized to study the stability of the particles system. The higher the absolute value of zeta potential, the greater the electrostatic repulsion between particles, and the more stable was the system. For example, particles have good stability when the absolute value of zeta potential is greater than 60 mV. On the contrary, when the absolute value of zeta potential is less than 10 mV, the particles will experience agglomerate or accumulate quickly (29). As shown in Figure 1C, the absolute value of zeta potential of S/O/W calcium-lipid emulsions increased from 39.71 ± 2.87 mV to 45.91 ± 0.97 mV with the increase in the concentration of PGA-XG complexes (0.4–0.8 wt%). Although their absolute value of zeta potential increased, S/O/W calcium-lipid emulsions presented negative values in all PGA-XG complexes concentration environments because XG is an anionic extracellular polysaccharide. This was due to the increase in the net charge number of S/O/W calcium-lipid emulsions with the increase in PGA-XG complexes concentration, which increased the electrostatic repulsion between the droplets. It could effectively prevent the aggregation of the droplets and increase the stability of S/O/W calcium-lipid emulsions. As stated above, this was consistent with the average particle size in Figure 1A.

### Physical stability of S/O/W calcium-lipid emulsions

**Figure 2:**
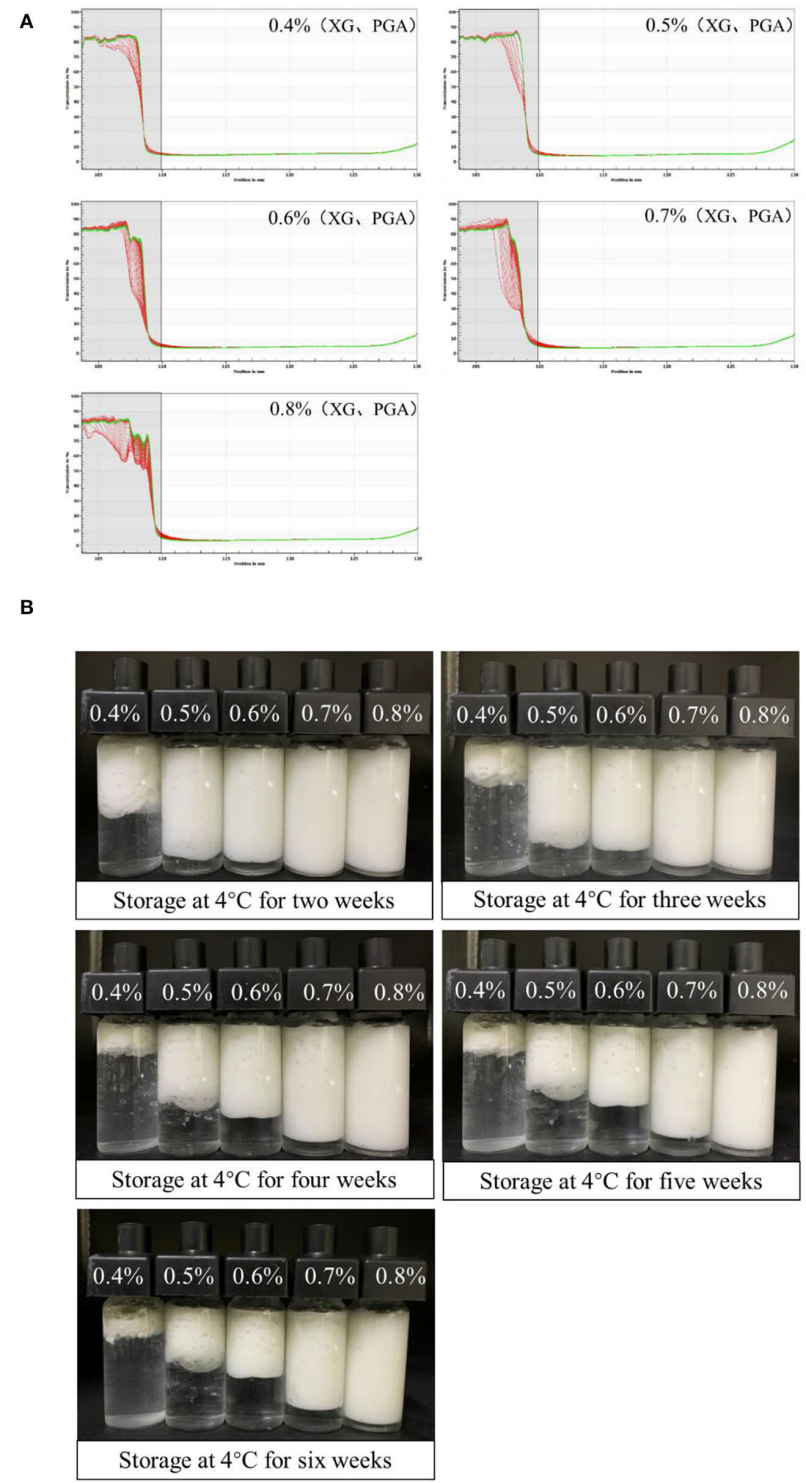
Physical stability tested by LUMiSizer [**(A)**, original transmission] of PGA-XG complexes stabilized S/O/W calcium-lipid emulsions as a function of concentrations. All the first profiles lie at the bottom, the last profiles lie at the top. Visual images **(B)** of the creaming stability of Solid-in-Oil-in-Water (S/O/W) calcium-lipid emulsions stabilized by different concentrations of PGA-XG complexes (0.4, 0.5, 0.6, 0.7, and 0.8 wt%) during 42 days of storage at 4°C.

The stability of S/O/W calcium-lipid emulsions was reflected by the particle migration rate (30). Only uniform S/O phase can form stable S/O/W calcium-lipid emulsions. In our previous study, we screened a series of the ratios of the S/O phase with the W phase and found that the CaCO_3_ has good dispersibility and stability when the proportion of S/O phase in S/O/W calcium-lipid emulsions was 5% (24). Therefore, the physical stability of S/O/W calcium-lipid emulsions as a function of the concentration of PGA-XG complexes was detected by LUMiSizer measurements. The stability was expressed as the evolution of the integrated transmission–time profiles determined at 4,000 rpm over 4 h 15 min at 25°C. The position of about 110 mm in the transmission profile corresponded to the filling height of the S/O/W calcium-lipid emulsions. The bottom position of the cell was 130 mm. The transmission curve represented the change of the droplet concentration in S/O/W calcium-lipid emulsions. All the first profiles lay at the bottom, and the last profiles lay at the top. In Figure 2A, the particle migration rate of all samples was small, whereas the speed of the interfacial movement decreases with the increase in the concentration of PGA-XG complexes. This was because the viscosity increases with the increase in the concentration of PGA-XG complexes. This phenomenon can also be reflected by the instability index which is the integrated transmission profiles against the measuring time. The instability index of S/O/W calcium-lipid emulsions with different concentrations of PGA-XG complexes is shown in Figure 1D. As can be seen, the order of instability index was: 0.4 > 0.5 > 0.6 > 0.7 > 0.8 wt% PGA-XG complexes concentration prepared S/O/W calcium-lipid emulsions. The reason may be that when the ratio of the S/O phase with the W phase was constant, the physical stability of the S/O/W calcium-lipid emulsions mainly depends on the type and concentration of the W phase (31).

Figure 2B shows the appearance of S/O/W calcium-lipid emulsions after 2, 3, 4, 5, and 6 weeks of storage at 4°C. The freshly prepared S/O/W calcium-lipid emulsions were all milky white, without any signs of separation or creaming. After 2 weeks of storage, there was a clear phase separation in the S/O/W calcium-lipid emulsions of 0.4 wt% PGA-XG complexes concentration, and a transparent aqueous phase appeared in the lower part. With the extension of storage time, this phenomenon also occurred in varying degrees of S/O/W calcium-lipid emulsions with 0.5, 0.6, and 0.7 wt% concentrations of PGA-XG complexes, while 0.8 wt% was not obvious, and still maintained excellent stability. This shows that 0.8 wt% concentrations of PGA-XG complexes can be better wrapped around small-size oil droplets to prevent the aggregation of adjacent droplets to produce creaming. Therefore, in all samples under visual observation, the S/O/W calcium-lipid emulsion with 0.8 wt% concentration of PGA-XG complexes, as emulsifier, has good storage stability. The results were consistent with the changes in the average particle size, zeta potential, and instability index.

### Apparent viscosity measurement

**Figure 3:**
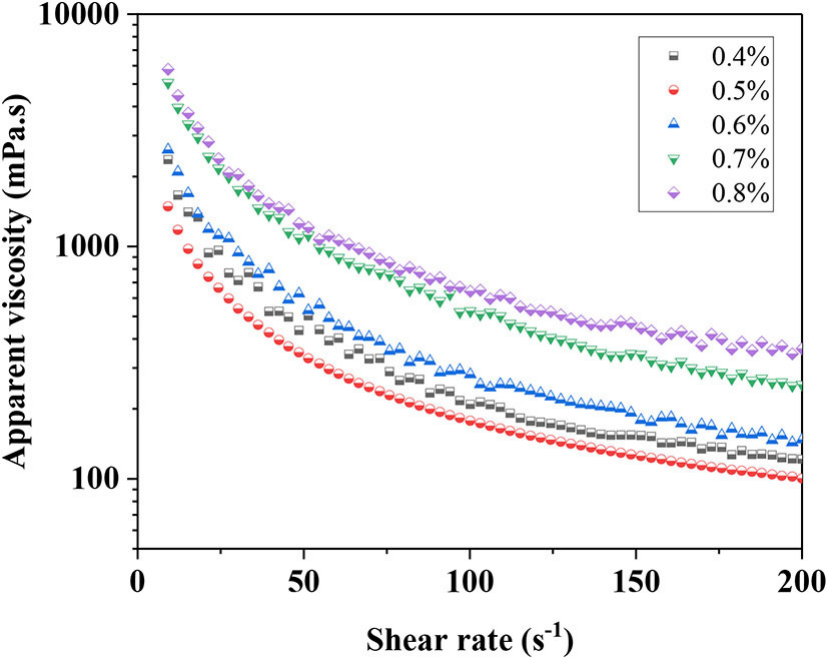
Apparent viscosity of S/O/W calcium-lipid emulsions at different concentrations of PGA-XG complexes (0.4, 0.5, 0.6, 0.7, and 0.8 wt%) determined by rotation rheometer.

The rheological properties of emulsions were particularly important for some applications (32). The apparent viscosity of emulsions is a part of rheological properties and is closely related to the stability of the emulsion. For instance, the aggregation and coalescence of oil droplets in S/O/W calcium-lipid emulsions to a large extent depend on the viscosity of the continuous phase. The apparent viscosity curve of S/O/W calcium-lipid emulsions was determined by setting a shear rate ramps from 1 to 200 s^−1^ at 25°C. Figure 3 shows that the apparent viscosity of all samples gradually decreased as the shear rate increased from 1 to 200 s^−1^, reflecting the progressive shear-induced breakdown of structure in the S/O/W calcium-lipid emulsions and the thixoplastic pseudoplastic rheological behavior of the S/O/W calcium-lipid emulsions. This was because when applied high shear stress, the S/O/W calcium-lipid emulsions cause strong elongation, network fracture, and droplet deformation (33). The chains between the PGA-XG complexes become loose and the interaction between molecules weakens. As expected, the viscosity of the S/O/W calcium-lipid emulsions increased with the increasing concentration of PGA-XG complexes. With the same shear rate conditions, the S/O/W calcium-lipid emulsion of 0.8 wt% concentrations of PGA-XG complexes showed the highest apparent viscosity. This was due to the formation of hydrogen bonds between XG molecules and water molecules in PGA-XG complexes, forming a network structure with thickening properties. With the increase in the concentration of PGA-XG complexes, the XG content increased, and the viscosity of the emulsion increased. Meanwhile, this type of thixoplastic pseudoplastic rheological behavior suggests that there may have been flocculated particles within the S/O/W calcium-lipid emulsions under quiescent conditions that were disrupted by shearing, which is consistent with the physical stability and average particle size in the previous section. The consistency coefficient (k) was the index of emulsion viscosity and combined with the visual images in Figure 2B, the change in the apparent viscosity showed a similar trend with the consistency coefficient of the compound system.

### Microstructure characteristics

**Figure 4:**
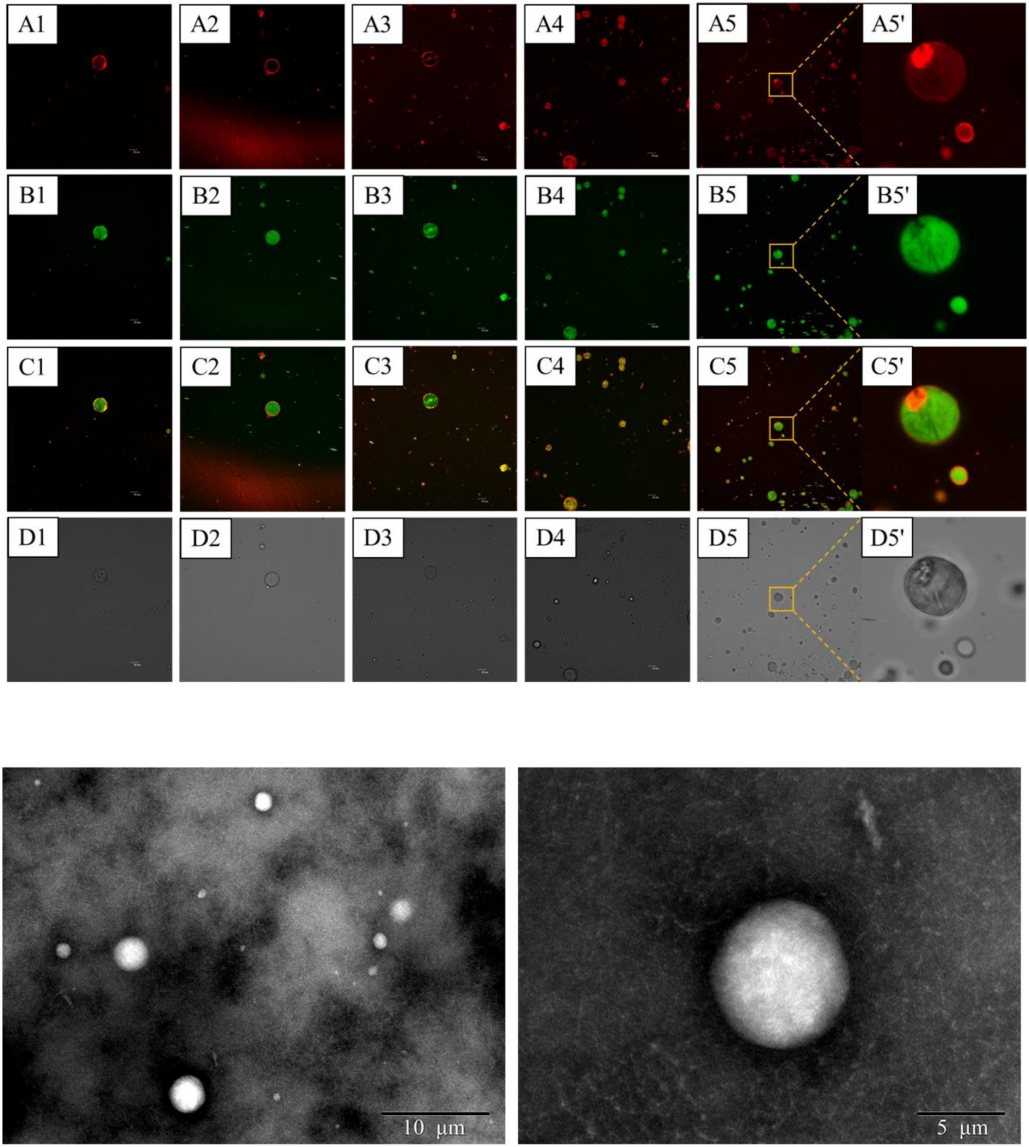
Confocal laser scanning microscope (CLSM) images **(A)** of S/O/W calcium-lipid emulsions stabilized by different concentrations of PGA-XG complexes. The calcium carbonate (CaCO_3)_ dyed by Rhodamine B (RB) (excitation at 543 nm) showed red fluorescence **(A)**, whereas the oil droplet dyed by Nile Red (excitation at 488 nm) showed green fluorescence **(B)**. Combined image **(C)**. Bright field **(D)**. The scale bar is 10 μm (1–5: 0.4, 0.5, 0.6, 0.7, and 0.8 wt%, respectively; 5': high magnified image at 0.8 wt%). Transmission electron microscopy (TEM) images **(B)** of S/O/W calcium-lipid emulsions stabilized by 0.8 wt% concentration of PGA-XG complexes.

Confocal laser scanning microscopy observation was conducted as a direct method to monitor the distribution of CaCO_3_ of the S/O/W calcium-lipid emulsions. Thus, to gain insight into such a unique microstructure of the S/O/W calcium-lipid emulsions and the distribution of CaCO_3_, we visualized the S/O/W calcium-lipid emulsions under CLSM. The CLSM images of S/O/W calcium-lipid emulsions are exhibited in Figure 4A. In the CLSM images, the red fluorescence (A1-5,5′) identified the RB-stained CaCO_3_, the green fluorescence (B1-5,5′) exhibited the Nile Red-dyed oil droplets, and the overlapped fluorescence (C1-5,5′) of RB (red fluorescence) and Nile Red (green fluorescence) evidently showed the encapsulation of many CaCO_3_ particles in O phase and the attachment of some CaCO_3_ particles at the boundary of the O phase. In addition, the darkness in the O phase may be caused by CaCO_3_ in other sections of the S/O/W calcium-lipid microspheres, which also showed that CaCO_3_ was encapsulated in the O phase. Similar microstructures in anthocyanin encapsulation emulsion have been reported in the literature (34). CLSM images confirmed the formation of S/O/W calcium-lipid microspheres. It could be observed that all the S/O/W calcium-lipid emulsion samples stabilized by the PGA-XG complexes significantly displayed smaller microspheres size and narrow polydispersity with increasing concentration of PGA-XG complexes. The reason for this phenomenon was that the high viscosity and strong electrostatic repulsion produced with the increase in the concentration of PGA-XG complexes can hinder the accumulation between adjacent microspheres. The light scattering result was consistent with these results, which were complementary data for the average particle size and particle size distribution of the S/O/W calcium-lipid emulsions analysis. Similar variation trends in the microsphere size vs. gum concentration have been reported in the previous study (35). The TEM images of S/O/W calcium-lipid emulsions are exhibited in Figure 4B. Figure 4B shows that the S/O/W calcium-lipid emulsion presents a white microsphere, while the outside of this microsphere is coated by PGA-XG complexes. This is consistent with the results observed by CLSM.

### X-ray diffraction analysis

**Figure 5:**
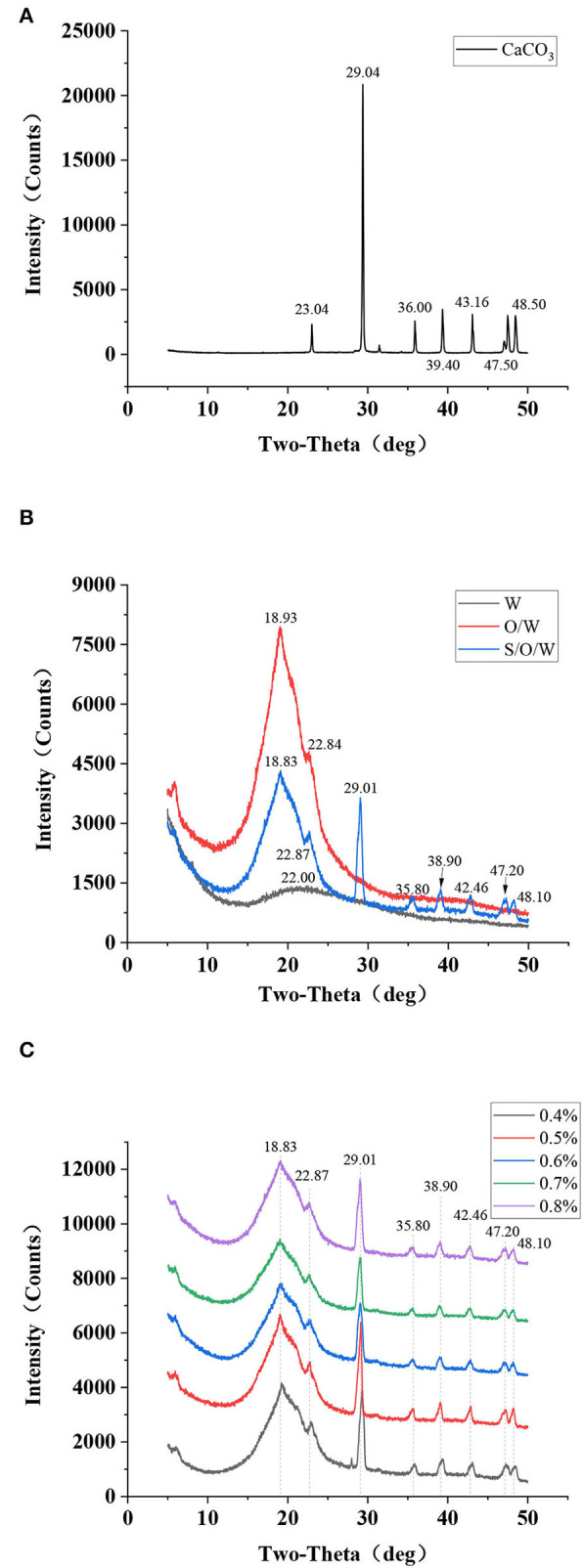
X-ray diffraction (XRD) patterns of **(A)** CaCO_3_ powders, **(B)** PGA-XG complexes, O/W emulsion without CaCO_3_, and S/O/W calcium-lipid emulsion, and **(C)** S/O/W calcium-lipid emulsions formed by different concentrations of PGA-XG complexes.

X-ray diffraction technology is a modern analytical method for testing solid powder samples. The crystal plane spacing, and the relative intensity diffracted in the XRD pattern can reflect the inherent properties of the material and analyze the phase composition and crystal type of the mineral. Jiang et al. (36) detected a degradation layer containing CaCO_3_ on the magnesium surface by XRD. Thus, to investigate the encapsulation of CaCO_3_ in the S/O/W calcium-lipid emulsions and its possible effect on the molecular interaction of the O phase and W phase, XRD data have been acquired. The X-ray spectra of CaCO_3_ in Figure 5A manifests that CaCO_3_ had feature crystalline peaks at 2θ diffraction angles of 23.04, 29.04, 36.00, 39.40, 43.16, 47.50, and 48.50°, corresponding to (012), (104), (110), (113), (202), (018), and (116) crystal planes, respectively. These feature crystalline peaks are consistent with those reported in the previous study (37), indicating that they have calcite structure. Figure 5B shows the XRD patterns of the PGA-XG complexes, O/W emulsion without CaCO_3_, and S/O/W calcium-lipid emulsion. As can be observed, the feature crystalline peaks of W phase around 22°, the feature crystalline peaks of O/W emulsion without CaCO_3_ at 18.93° and 22.84°, the feature crystalline peaks of S/O/W calcium-lipid emulsion at 18.83, 22.87, 29.01, 35.80, 38.90, 42.46, 47.20, and 48.10°. Compared with the diffraction pattern of O/W emulsion without CaCO_3_, the S/O/W calcium-lipid emulsion diffraction diagram showed crystallization peaks at 29.01, 35.80, 38.90, 42.46, 47.20, and 48.10°, which was caused by the diffraction peaks of CaCO_3_ at the above angles. Compared with the diffraction pattern of CaCO_3_, the S/O/W calcium-lipid emulsion feature crystalline peaks widened and the peak intensity weakened obviously. This result respectively demonstrates that the particle size decreases and the O/W emulsion was involved in the crystallization process of CaCO_3_, that is, CaCO_3_ was well dispersed in the emulsion system, which reduced the intermolecular force of CaCO_3_, thereby reducing the crystallization ability and the crystallization area of CaCO_3_ (38). The curves in Figure 5C show the XRD diagram of S/O/W calcium-lipid emulsions formed by different concentrations of PGA-XG complexes. It can be seen that the diffraction peaks are similar, indicating that the grain composition is the same. Observation of comparable amorphous structures has already been reported in the literature (25, 39).

### Fourier transform infrared spectroscopy measurement

**Figure 6:**
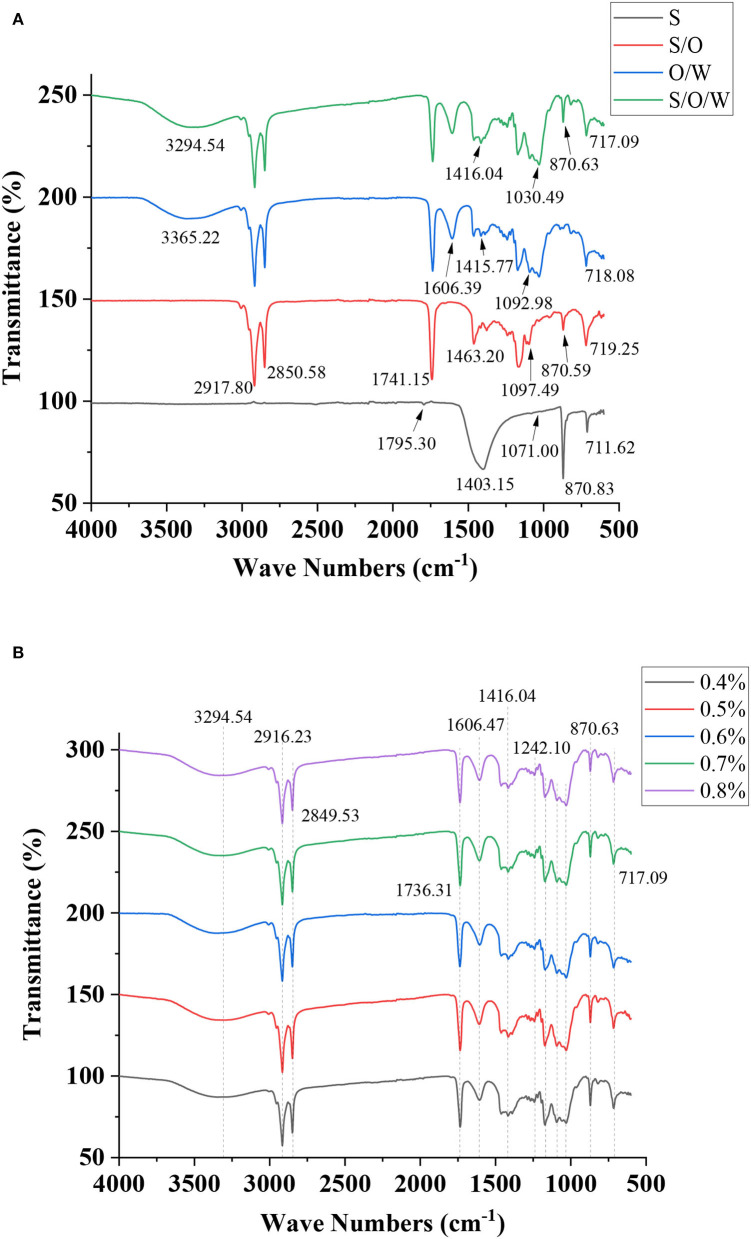
Fourier transform infrared (FTIR) spectra of **(A)** CaCO_3_ powders, S/O phase, O/W emulsion without CaCO_3_, S/O/W calcium-lipid emulsion, and **(B)** S/O/W calcium-lipid emulsions formed by different concentrations of PGA-XG complexes.

Fourier transform infrared spectroscopy is an effective method for characterizing the molecular interactions within different components. The FTIR spectra of CaCO_3_ powders, S/O phase, O/W emulsion without CaCO_3_, and S/O/W calcium-lipid emulsion were analyzed and illustrated in Figure 6A. The FTIR spectrum of the CaCO_3_ powders showed vibration frequency bands at 1795.30, 1403.15, 1071.00, 870.83, and 711.62 cm^−1^, due to C = O stretching vibration, C-O asymmetric stretching vibration, C-O symmetric stretching vibration, C-O in-plane bending vibration, and C-O out-of-plane bending vibration, respectively. The S/O phase showed relatively characteristic peaks at 2,917.80, 2,850.58, 1,741.15, 1,463.20, 1,097.49, 870.59, and 719.25 cm^−1^. One can see that the absorption region of the S/O phase at 1,403.15, 870.83, and 711.62 cm^−1^ are weaker than that of CaCO_3_ powders. Such a difference was likely due to the small amount of CaCO_3_ on the oil surface observed by the CLSM [Figure 4 (C5')]. The characteristic absorption peaks of O/W emulsion without CaCO_3_ appeared at 3,365.22, 2,916.31, 2,849.59, 1,736.51, 1,606.39, 1,463.65, 1,415.77, 1,092.98, 1,030.83, 890.12, and 718.08 cm^−1^. In the high-frequency region of the S/O/W calcium-lipid emulsion spectrum, the wavenumber at 3,294.54 cm^−1^ was assigned as -OH, while C-H stretching and bending vibration was observed at 2,916.23 cm^−1^. The bands at 1,736.31 cm^−1^ can be attributed to the C = O stretching in the acetyl groups, the one at 1,606.47 cm^−1^ to the intramolecular hydrogen bonds, and those at 1,000 to 1,200 cm^−1^ to the C-O-H, C-C, and C-O-C. Additionally, bands appearing at 870.63 and 717.09 cm^−1^ are characteristic of mannose in PGA-XG complexes, as reported for emulsions stabilized by polysaccharide complex (40). Compared with the spectrum of O/W emulsion without CaCO_3_, the peaks of S/O/W calcium-lipid emulsion caused a redshift at 870.63 cm^−1^, which was the C-O in-plane bending vibration induced by CaCO_3._ In the S/O/W calcium-lipid emulsion, the characteristic peak of CaCO_3_ at 1403.15 cm^−1^ was not observable, which indicates that CaCO_3_ was well embedded in the emulsion system. The results are consistent with the CLSM in the previous chapter. Additionally, as shown in Figure 6B, the characteristic absorption peaks of S/O/W calcium-lipid emulsions formed by different concentrations of PGA-XG complexes are similar, indicating that there is no difference in the group formation of all samples, and the interaction is consistent.

### Raman spectrum analysis

**Figure 7:**
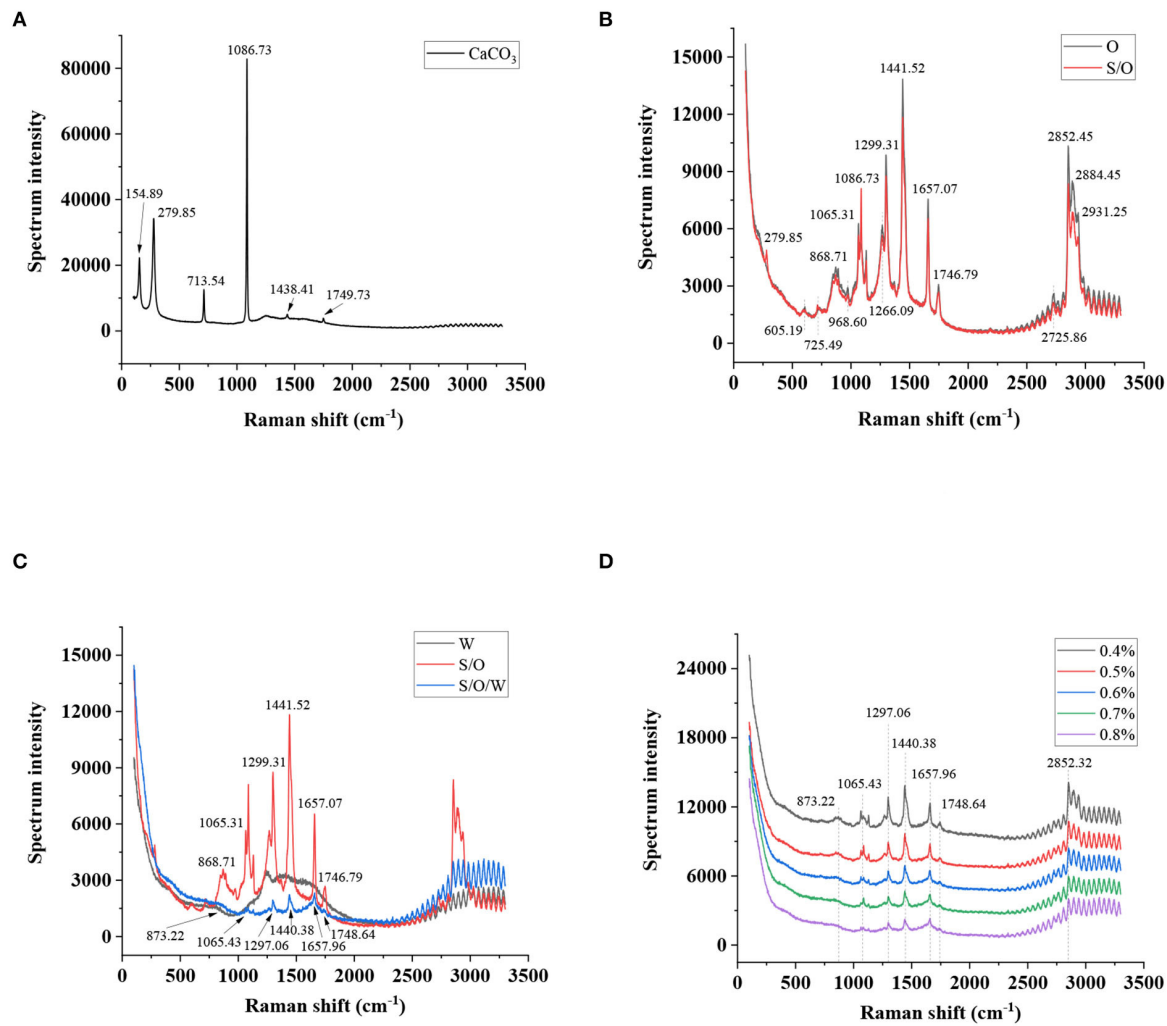
Raman spectra of **(A)** CaCO_3_ powders, **(B)** O phase and S/O phase, **(C)** W phase, S/O phase, and S/O/W calcium-lipid emulsion, and **(D)** S/O/W calcium-lipid emulsions formed by different concentrations of PGA-XG complexes.

Raman spectroscopy can provide molecular structure information of different functional groups to characterize the interaction of emulsion components (41). Raman spectrum of CaCO_3_ powders is presented in Figure 7A. Significant characteristic peaks were observed at 1,749.73, 1,438.41, 1,086.73, 713.54, 279.85, and 154.89 cm^−1^ in the Raman spectra of CaCO_3_ powders. The result was also reported by Gunasekaran (42). As shown in Figure 7B, the Raman spectra of the O phase and S/O phase were analyzed. In the spectra of O phase, the vibrational bands at 605.19 cm^−1^ (ring deformations), 725.49 cm^−1^ (symmetric and asymmetric stretching vibrations of the choline N^+^(CH_3_)_3_ group), 868.71 cm^−1^ (phospholipids), 968.60 cm^−1^ (C = C bending), 1,065.31 cm^−1^ (C-C stretching vibrations), 1,266.09 cm^−1^ (=CH bending, scissoring), 1,299.31 cm^−1^ (C-H twisting vibrations), 1,441.52 cm^−1^ (C-H bending, scissoring), 1,657.07 cm^−1^ (C = C stretching vibrations), 1,746.79 cm^−1^ (RC = OOR, C = O stretching), 2,725.86 cm^−1^ (C-H stretching vibrations), 2,852.45 cm^−1^ (C-H symmetric stretching vibrations of CH_2_ group), 2,884.45 cm^−1^ (C-H symmetric stretching vibrations of CH_3_ group), and 2,931.25 cm^−1^ (C-H asymmetric stretching vibrations of CH_2_ group) were consistent with those reported in the literature (43, 44). In the spectrum of the S/O phase, the strong characteristic peak of CaCO_3_ powders at 1,086.73 cm^−1^ was greatly weakened, indicating that most CaCO_3_ was inside the O phase, which proved the formation of the S/O phase. Overlapped spectra of the W phase, S/O phase, and S/O/W calcium-lipid emulsions are presented in Figure 7C. The characteristic peaks of the W phase spectrum are around 1,200 to 1,750 cm^−1^, which is due to the saccharides, pyruvate, and acetyl of XG (45). Compared with the S/O phase spectra, the characteristic peaks at 873.22, 1,065.43, 1,297.06, 1,440.38, 1,657.96, and 1,748.64 cm^−1^ of the S/O/W calcium-lipid emulsions were almost not observable, which were assigned to the formation of the interface layer of W phase on S/O phase surface. The Raman spectra of S/O/W calcium-lipid emulsions formed by different concentrations of PGA-XG complexes are shown in Figure 7D, which shows that the intensity of the peaks was at 873.22, 1,065.43, 1,297.06, 1,440.38, 1,657.96, 1,748.64, and 2,852.32 cm^−1^, and the intensity slightly decreased with the rising concentrations of PGA-XG complexes (0.4, 0.5, 0.6, 0.7, and 0.8 wt%) in S/O/W calcium-lipid emulsions, indicating that the interfacial film became thicker with the increase of W phase, and the embedding effect was enhanced. The above results show that the S/O/W calcium-lipid emulsion was formed.

## Conclusion

A type of emulsion to load CaCO_3_ powders using PGA-XG complexes based on S/O/W techniques was fabricated. Our findings showed that when the concentration of PGA-XG complexes was 0.8 wt%, the S/O/W calcium-lipid emulsion exhibited smaller particle size, higher absolute value of zeta potential, greater apparent viscosity, and improved physical stability. CLSM images and TEM images confirmed that the CaCO_3_ powders encapsulated on the O phase, forming S/O/W calcium-lipid microspheres. The XRD analysis further confirmed that CaCO_3_ was loaded in the S/O/W calcium-lipid emulsion as an amorphous state. The FTIR and Raman spectrum analysis indicated that the emulsion layer was formed at solid-oil interface and oil-water interface. This S/O/W calcium-lipid emulsion design can overcome the defect of the poor solubility of CaCO_3_. This study contributes to the development of the formation of mineral elements loaded with microspheres based on S/O/W techniques. Additionally, it may also accelerate the creation of a novel type of calcium preparation with achieved higher quality utilization.

## Data availability statement

The original contributions presented in the study are included in the article/supplementary material, further inquiries can be directed to the corresponding author/s.

## Author contributions

GL: writing—review and editing, methodology, data curation, and writing—original draft. YZ: methodology and investigation. JZ: formal analysis and helped perform the analysis with constructive discussions. JH: validation. DX: conceptualization, project administration, and supervision. YC: project administration and funding acquisition. All authors contributed to the article and approved the submitted version.

## Funding

This work was supported by the National Natural Science Foundation of China (31871808).

## Conflict of interest

The authors declare that the research was conducted in the absence of any commercial or financial relationships that could be construed as a potential conflict of interest.

## Publisher's note

All claims expressed in this article are solely those of the authors and do not necessarily represent those of their affiliated organizations, or those of the publisher, the editors and the reviewers. Any product that may be evaluated in this article, or claim that may be made by its manufacturer, is not guaranteed or endorsed by the publisher.

## References

[1] Widdowson EM Dickerson JW. The effect of growth and function on the chemical composition of soft tissues. Biochem J. (1960) 77:30–43. 10.1042/bj0770030 13785015 PMC1204895

[2] Balk EM Adam GP Langberg VN Earley A Clark P Ebeling PR. Global dietary calcium intake among adults: a systematic review. Osteoporosis Int. (2017) 28:3315–24. 10.1007/s00198-017-4230-x 29026938 PMC5684325

[3] Elsheikh M Kishida R Hayashi K Tsuchiya A Shimabukuro M Ishikawa K. Effects of pore interconnectivity on bone regeneration in carbonate apatite blocks. Regen Biomater. (2022) 9:rbac010. 10.1093/rb/rbac010 35449826 PMC9017375

[4] Meng D Dong L Yuan Y Jiang Q. In vitro and in vivo analysis of the biocompatibility of two novel and injectable calcium phosphate cements. Regen Biomater. (2019) 6:13–9. 10.1093/rb/rby027 30740238 PMC6362821

[5] Chen Z Lv X Zhao M Zhang P Ren X Mei X. Encapsulation of green tea polyphenol by pH responsive, antibacterial, alginate microgels used for minimally invasive treatment of bone infection. Colloid Surf B-Biointerfaces. (2018) 170:648–55. 10.1016/j.colsurfb.2018.06.065 29986260

[6] Weaver CM Heaney RP Nickel KP Packard PI. Calcium bioavailability from high oxalate vegetables: Chinese vegetables, sweet potatoes and rhubarb. J Food Sci. (1997) 62:524–5. 10.1111/j.1365-2621.1997.tb04421.x

[7] Kopic S Geibel JP. Gastric acid, calcium absorption, and their impact on bone health. Physiol Rev. (2013) 93:189–268. 10.1152/physrev.00015.2012 23303909

[8] Raikos V Ranawana V. Designing emulsion droplets of foods and beverages to enhance delivery of lipophilic bioactive components - a review of recent advances. Int J Food Sci Technol. (2017) 52:68–80. 10.1111/ijfs.13272

[9] Abaee A Mohammadian M Jafari SM. Whey and soy protein-based hydrogels and nano-hydrogels as bioactive delivery systems. Trends Food Sci Technol. (2017) 70:69–81. 10.1016/j.tifs.2017.10.011

[10] McClements DJ. Enhancing nutraceutical bioavailability through food matrix design. Curr Opin Food Sci. (2015) 4:1–6. 10.1016/j.cofs.2014.12.008

[11] Zhang R Han Y Xie W Liu F Chen S. Advances in protein-based nanocarriers of bioactive compounds: from microscopic molecular principles to macroscopical structural and functional attributes. J Agric Food Chem. (2022) 70:6354–67. 10.1021/acs.jafc.2c01936 35603429

[12] Torres O Murray B Sarkar A. Emulsion microgel particles: novel encapsulation strategy for lipophilic molecules. Trends Food Sci Technol. (2016) 55:98–108. 10.1016/j.tifs.2016.07.006

[13] McIntyre I O'Sullivan M O'Riordan D. Monitoring the progression of calcium and protein solubilisation as affected by calcium chelators during small-scale manufacture of casein-based food matrices. Food Chem. (2017) 237:597–604. 10.1016/j.foodchem.2017.05.149 28764041

[14] Wu J Zhong Q. Encapsulation of konjac glucomannan in oil droplets to reduce viscosity of aqueous suspensions and gradually increase viscosity during simulated gastric digestion. J Food Eng. (2016) 175:104–7. 10.1016/j.jfoodeng.2015.12.010

[15] Zhang Y Lin J Zhong Q. S/O/W emulsions prepared with sugar beet pectin to enhance the viability of probiotic Lactobacillus salivarius NRRL B-30514. Food Hydrocoll. (2016) 52:804–10. 10.1016/j.foodhyd.2015.08.020

[16] Zhang Y Zhong Q. Freeze-dried capsules prepared from emulsions with encapsulated lactase as a potential delivery system to control lactose hydrolysis in milk. Food Chem. (2018) 241:397–402. 10.1016/j.foodchem.2017.09.004 28958545

[17] Morita T Sakamura Y Horikiri Y Suzuki T Yoshino H. Protein encapsulation into biodegradable microspheres by a novel S/O/W emulsion method using poly(ethylene glycol) as a protein micronization adjuvant. J Control Release. (2000) 69:435–44. 10.1016/S0168-3659(00)00326-6 11102683

[18] Kurt A Toker OS Tornuk F. Effect of xanthan and locust bean gum synergistic interaction on characteristics of biodegradable edible film. Int J Biol Macromol. (2017) 102:1035–44. 10.1016/j.ijbiomac.2017.04.081 28450249

[19] Garcia-Ochoa F Santos VE Casas JA Gomez E. Xanthan gum: production, recovery, and properties. Biotechnol Adv. (2000) 18:549–79. 10.1016/S0734-9750(00)00050-1 14538095

[20] Kool MM Schols HA Delahaije R Sworn G Wierenga PA Gruppen H. The influence of the primary and secondary xanthan structure on the enzymatic hydrolysis of the xanthan backbone. Carbohydr Polym. (2013) 97:368–75. 10.1016/j.carbpol.2013.05.045 23911459

[21] Cheong KW Mirhosseini H Hamid NSA Osman A Basri M Tan CP. Effects of propylene glycol alginate and sucrose esters on the physicochemical properties of modified starch-stabilized beverage emulsions. Molecules. (2014) 19:8691–706. 10.3390/molecules19068691 24962400 PMC6270833

[22] Chen YC Chen CC Hsieh JF. Propylene glycol alginate-induced coacervation of milk proteins: A proteomics approach. Food Hydrocoll. (2016) 53:233–8. 10.1016/j.foodhyd.2015.01.018

[23] Wang D Mao L Dai L Yuan F Gao Y. Characterization of chitosan-ferulic acid conjugates and their application in the design of beta-carotene bilayer emulsions with propylene glycol alginate. Food Hydrocoll. (2018) 80:281–91. 10.1016/j.foodhyd.2017.11.031

[24] Zhang J Li G Xu D Cao Y. Stability, microstructure, and rheological properties of CaCO[[sb]]3[[/s]] S/O/W Calcium-Lipid Emulsions. Foods. (2021) 10:2216. 10.3390/foods10092216 34574326 PMC8468493

[25] Xu D Gao Q Ma N Hao J Yuan Y Zhang M. Structures and physicochemical characterization of enzyme extracted oil bodies from rice bran. LWT-Food Sci Technol. (2021) 135:109982. 10.1016/j.lwt.2020.109982

[26] Guo X Li X Chan L Huang W Chen T. Edible CaCO[[sb]]3[[/s]] nanoparticles stabilized Pickering emulsion as calcium-fortified formulation. J Nanobiotechnol. (2021) 19:67. 10.1186/s12951-021-00807-6 33663532 PMC7934247

[27] Juttulapa M Piriyaprasarth S Takeuchi H Sriamornsak P. Effect of high-pressure homogenization on stability of emulsions containing zein and pectin. Asian J Pharm Sci. (2017) 12:21–7. 10.1016/j.ajps.2016.09.004 32104310 PMC7032127

[28] Ohshima H. Interaction of colloidal particles, Colloid and Interface Science in Pharmaceutical Research and Development. (2014) p. 1–28. 10.1016/B978-0-444-62614-1.00001-6

[29] Yu W Xie H. A review on nanofluids: preparation, stability mechanisms, and applications. J Nanomater. (2012) 2012:1–17. 10.1155/2012/435873

[30] Lu X Chen J Zheng M Guo J Qi J Chen Y. Effect of high-intensity ultrasound irradiation on the stability and structural features of coconut-grain milk composite systems utilizing maize kernels and starch with different amylose contents. Ultrason Sonochem. (2019) 55:135–48. 10.1016/j.ultsonch.2019.03.003 30853534

[31] Tian H Xiang D Li C. Tea polyphenols encapsulated in W/O/W emulsions with xanthan gum-locust bean gum mixture: Evaluation of their stability and protection. Int J Biol Macromol. (2021) 175:40–8. 10.1016/j.ijbiomac.2021.01.161 33548306

[32] McClements DJ. Critical review of techniques and methodologies for characterization of emulsion stability. Crit Rev Food Sci Nutr. (2007) 47:611–49. 10.1080/10408390701289292 17943495

[33] Yildirim M Sumnu G Sahin S. The effects of emulsifier type, phase ratio, and homogenization methods on stability of the double emulsion. J Dispersion Sci Technol. (2016) 38:807–14. 10.1080/01932691.2016.1201768

[34] Xiao J Lu X Huang Q. Double emulsion derived from kafirin nanoparticles stabilized Pickering emulsion: Fabrication, microstructure, stability and in vitro digestion profile. Food Hydrocoll. (2017) 62:230–8. 10.1016/j.foodhyd.2016.08.014

[35] Khouryieh H Puli G Williams K Aramouni F. Effects of xanthan-locust bean gum mixtures on the physicochemical properties and oxidative stability of whey protein stabilised oil-in-water emulsions. Food Chem. (2015) 167:340–8. 10.1016/j.foodchem.2014.07.009 25148996

[36] Jiang W Lin J Chen AH Pan J Liu H. A portable device for studying the effects of fluid flow on degradation properties of biomaterials inside cell incubators. Regen Biomater. (2019) 6:39–48. 10.1093/rb/rby026 30740241 PMC6362820

[37] Kontoyannis CG Vagenas NV. Calcium carbonate phase analysis using XRD and FT-Raman spectroscopy. Analyst. (2000) 125:251–5. 10.1039/a908609i

[38] Bolívar G Colina M Delgado B Mendizabal E. The effect of carboxymethyl chitosan on calcium carbonate precipitation in synthetic brines. J Mex Chem Soc. (2021) 65:109–17. 10.29356/jmcs.v65i1.1429

[39] Hao J Xu J Zhang W Li X Liang D Xu D. The improvement of the physicochemical properties and bioaccessibility of lutein microparticles by electrostatic complexation. Food Hydrocoll. (2022) 125:107381. 10.1016/j.foodhyd.2021.107381

[40] Li Y Xiang D Wang B Gong X. Oil-in-water emulsions stabilized by ultrasonic degraded polysaccharide complex. Molecules. (2019) 24:1097. 10.3390/molecules24061097 30897726 PMC6471402

[41] Samyn P Van Nieuwkerke D Schoukens G Vonck L Stanssens D Van Den Abbeele H. Quality and statistical classification of brazilian vegetable oils using mid-infrared and raman spectroscopy. Appl Spectrosc. (2012) 66:552–65. 10.1366/11-06484 22524961

[42] Gunasekaran S Anbalagan G. Spectroscopic characterization of natural calcite minerals. Spectroc Acta Pt A-Molec Biomolec Spectr. (2007) 68:656–64. 10.1016/j.saa.2006.12.043 17331794

[43] Taylan O Cebi N Tahsin Yilmaz M Sagdic O Bakhsh AA. Detection of lard in butter using Raman spectroscopy combined with chemometrics. Food Chem. (2020) 332:127344. 10.1016/j.foodchem.2020.127344 32619937

[44] Lee JY Park JH Mun H Shim WB Lim SH Kim MG. Quantitative analysis of lard in animal fat mixture using visible Raman spectroscopy. Food Chem. (2018) 254:109–14. 10.1016/j.foodchem.2018.01.185 29548429

[45] Sampaio ICF Crugeira PJL Soares LGP Dos Santos JN de Almeida PF Pinheiro ALB. Composition of Xanthan gum produced by Xanthomonas campestris using produced water from a carbonated oil field through Raman spectroscopy. J Photochem Photobiol B-Biol. (2020) 213:112052. 10.1016/j.jphotobiol.2020.112052 33074141

